# Surveillance Strategy for Patients With BCLC Stage B Hepatocellular Carcinoma After Achieving Complete Remission: Data From the Real World

**DOI:** 10.3389/fonc.2020.574804

**Published:** 2020-09-29

**Authors:** Ying Wu, Lujun Shen, Han Qi, Fei Cao, Shuanggang Chen, Lin Xie, Tao Huang, Danyang Zhou, Jinqing Mo, Weijun Fan

**Affiliations:** ^1^Department of Minimally Invasive Interventional Therapy, Sun Yat-sen University Cancer Center, Guangzhou, China; ^2^State Key Laboratory of Oncology in South China, Collaborative Innovation Center of Cancer Medicine, Sun Yat-sen University, Guangzhou, China; ^3^Department of Medical Oncology, Sun Yat-sen University Cancer Center, Guangzhou, China

**Keywords:** hepatocellular carcinoma, BCLC stage B, surveillance interval, complete remission, extra-Milan criteria relapse

## Abstract

**Purpose:** There is a lack of consensus on the surveillance strategy for Barcelona Clinic liver cancer (BCLC) stage B hepatocellular carcinoma (HCC) patients with complete remission (CR). We performed a real-world, retrospective analysis of the surveillance strategy for BCLC stage B HCC patients after radical therapy with CR to support clinical decision-making.

**Materials and Methods:** We analyzed 546 BCLC stage B HCC patients with CR after radical treatments (surgery/ablation) at Sun Yat-sen University Cancer Center, from January 2007 to December 2019. The intensity of surveillance interval was defined as the mean of surveillance interval within 2 years. The primary endpoint of the study was overall survival (OS) and extra-Milan criteria relapse.

**Results:** During a median follow-up time of 23.9 months (range = 3.1–148.3 months), there were 11.9% of patients died, 56.6% of patients developed recurrence, the vast majority of patients experienced recurrence within 2 years, and 27.8% patients developed extra-Milan criteria recurrence. The median disease-free survival and OS were 33.6 and 60.0 months, respectively. Patients were divided into regular surveillance group (RS) (≤4.3 months) and irregular surveillance (IRS) group (>4.3 months) based on the optimal cutoff value of the intensity of surveillance interval. The RS group owned a lower incident of extra-Milan criteria relapse and smaller and fewer tumors at recurrence than IRS group, which contributed to the prolonged OS. Besides, the cutoff values of surveillance interval that could lead to significant differences in the incidence of extra-Milan criteria relapse during 0–6, 6–12, and 12–18 months after CR were 2.6, 2.9, and 3 months, respectively.

**Conclusions:** The average surveillance interval for patients with BCLC stage B HCC achieved CR should not exceed 4.3 months during the first 2 years' follow-up. During three different phases of the initial 18 months after CR, individualized surveillance showed intervals no more than 3 months were required to reduce the incidence of extra-Milan criteria relapse.

## Introduction

Hepatocellular carcinoma (HCC) is the sixth most commonly diagnosed cancer and the fourth leading cause of cancer death worldwide in 2018 (1). Rates of both incidence and mortality are two to three times higher among men in most regions (1). Hepatitis B virus (HBV) and hepatitis C virus (HCV) are considered to be the main pathogens for the development of HCC, especially in Asia (2). Recently, the number of patients with HCC originating from HCV has increased year by year, and the number of HCC patients owing to HBV has decreased (3).

The Barcelona Clinic liver cancer (BCLC) stage B (intermediate stage) (4) patients account for ~19.4% of total HCC (5). Also, the BCLC stage B represents a heterogeneous group of patients (6), which were more complicated and experience relapses earlier than BCLC stage A. The main factors were the span of liver function score (Child–Pugh: 5–9), the difference of tumor size (diameter 3–10 cm or more), tumor number (2–20 or more), and the difference of tumor distribution (single lobe limited or double lobe diffused).

Untreated patients at BCLC stage B present a median survival of 16 months or a survival rate of 49% at 2 years (7, 8). Chemoembolization extends the survival of these patients to a median of up to 19–20 months (8, 9). Surgery and ablation comprise potentially curative treatment modalities for BCLC stage B HCC patients (10). Besides, patients in this stage achieving downstaging from combined treatments or TACE (transarterial chemoembolization) can be suitable for radical treatments (6, 11). Unfortunately, the median survival of BCLC stage B HCC patients after curative treatment was 45 months (12). Tumor recurrence after curative surgery occurs in 50–70% of patients, which constitutes either intrahepatic metastases (often within 2 years after surgery) or a new HCC in the remaining cirrhotic liver (10, 13). Factors of early or late recurrence or/and metastases were complex, including tumor size and history of rupture, etc. (11, 14, 15).

Patients with recurrence after radical therapies may still be candidates for curative therapies (10, 16, 17). Early diagnosis of recurrence is more likely to receive curative treatment and achieve better disease control and prolonged survival (18). Although recent guidelines recommend surveillance strategies (10, 19) for patients after curative treatment, there is a lack of specific consensus on surveillance regimen after curative treatment of HCC, especially for BCLC stage B HCC patients with complete remission (CR) after radical treatment. For HCC patients with BCLC stage B, whether the current surveillance strategies are sufficient remains unclear. Moreover, although patients are recommended for surveillance according to the guidelines in the clinic, in the real world, for various reasons, patients cannot fully follow the guidelines for surveillance strategies. Therefore, the impact of irregular surveillance (IRS) in the real world on patient survival is also unclear.

Based on this background, we performed a real-world, retrospective analysis of the surveillance strategy for BCLC stage B HCC patients after radical therapy with CR to support clinical decision-making.

## Materials and Methods

### Patients

This study met the requirements of the Declaration of Helsinki and was approved by the Institutional Review Board of Sun Yat-sen University Cancer Center. We retrospectively analyzed BCLC stage B HCC patients who underwent radical therapy (surgery/ablation) from an institutional database at Sun Yat-sen University Cancer Center, from January 2007 to December 2019. A total of 2,193 consecutive patients were initially considered eligible. All cases were diagnosed as HCC according to pathology or clinical criteria (10, 19). This study included BCLC stage B HCC patients who received radical treatment (surgery/ablation) and achieved CR. Multidetector computed tomography (CT) and/or magnetic resonance imaging (MRI) were performed routinely to evaluate the local or distant extension of the primary tumors. Patients who visited our hospital at least 3 months after radical treatment were candidates for this study. CR is defined as no recurrence within 3 months after radical treatments. Patients were also excluded if they met any of the following criteria: age <18 or >75 years, non-HCC, mixed liver cancer, non-BCLC stage B, non-radical treatment, non-CR, died of postoperative complications. After excluding 1,637 patients according to the exclusion criteria, 546 patients were finally included in the study. All patients received radical treatment, including surgery and ablation. Some patients were treated with TACE before having undergone radical treatment, whereas others received a one-stage radical treatment.

### Surveillance Strategy

After radical operation, patients were informed to perform multiphasic, high-quality, cross-sectional imaging of the chest, abdomen, and pelvis every 3–6 months for 2 years and then followed up every 6–12 months as recommended by the guideline (19). Recurrence was defined as radiological evidence of intra-abdominal or abdominal soft tissue around the surgical site, or else distant metastasis. Besides, the date of each surveillance was recorded, and the end point of the surveillance was the time of tumor extra-Milan criteria recurrence and death. Intensity of surveillance interval was defined as the mean of surveillance interval within 2 years. For patients who died, survival time after curative treatment and the result of death were recorded.

### Statistical Analysis

Overall survival (OS) and extra-Milan criteria recurrence were measured from the date of CR to death or extra-Milan criteria recurrence or last follow-up evaluation. Continuous variables were presented as mean ± standard deviation and analyzed using the Student *t*-test. Categorical variables were analyzed using the χ^2^ or Fisher exact test, as appropriate. Survival rates were estimated by the Kaplan–Meier (K-M) method. Differences in OS were assessed for significance using the log-rank test. The Cox proportional hazards regression model was used to determine the factors associated with survival. As per initial design, all variables with a *P* < 0.05 by univariable analysis were entered in the multivariable analysis. Finally, only one variable was found to be associated with survival, and multivariable analysis could not be performed. Optimal cutoff for analysis was selected using X-Tile. Statistical analyses were performed using SPSS 18 for Windows (SPSS Inc., Chicago, IL, USA). Pictures were drawn using GraphPad Prism version 6.0 and R-3.6.3 software. All *P*-values were two-sided, and *P* < 0.05 was considered significant.

## Results

### Clinical and Pathological Characteristics of All HCC Patients

We enrolled 546 BCLC stage B HCC patients who achieved CR after radical treatment. The clinical and pathological characteristics of all the 546 patients are listed in Table 1. The 546 patients were followed 2,115 times, with an average of 4 times per person over 2 years. According to the best cutoff, regular surveillance group (RS) (*n* = 441) was defined as receipt of repeated CT/MRI with mean interval ≤4.3 months within 2 years. The IRS group (*n* = 105) was defined as receipt of repeated CT/MRI with mean interval >4.3 months within 2 years. Overall demographics were similar, but RS patients with a higher proportion of poor differentiation (*P* < 0.001).

**Table 1:** Correlation between surveillance interval and clinicopathological characteristics in HCC patients.

**Variable**		**RS (*****n*** **=** **441)**		**IRS (*****n*** **=** **105)**		***P***
		**%**	**No**.	**%**	**No**.	
Gender	Male	87.5	386	89.5	94	0.573
	Female	12.5	55	11.5	11	
Age (years)	≤41	18.8	83	11.4	12	0.073
	>41	81.2	358	88.6	93	
HBV or HCV	No	12.9	57	16.2	17	0.380
	Yes	87.1	384	83.8	88	
Cirrhosis	No	34.7	153	34.3	36	0.937
	Yes	65.3	288	65.7	69	
AFP (μg/L)	≤400	53.1	234	59.0	62	0.359
	>400	46.9	207	41.0	43	
Tumor size (mm)	≤40	38.1	168	37.1	39	0.857
	>40	61.9	273	62.9	66	
Multinodular tumor (≥4)	No	82.5	364	89.5	94	0.080
	Yes	17.5	77	10.5	11	
One-stage radical treatment	No	56.5	249	60.0	63	0.419
	Yes	43.5	192	40.0	42	
Therapeutic modalities	Surgery	78.9	348	83.8	88	0.261
	Ablation	21.1	93	16.2	17	
Differentiation	Well	4.2	15	13.2	12	**0.001**
	Moderated	53.1	188	60.4	55	
	Poor	42.7	151	26.4	24	
Satellite nodules	No	90.0	316	93.4	85	0.319
	Yes	10.0	35	6.6	6	
Venous invasion	No	67.2	236	73.6	67	0.240
	Yes	32.8	115	26.4	24	
Perineural invasion	No	99.7	350	97.8	89	0.109
	Yes	0.3	1	2.2	2	

*The meaning of the bold values provided was *p* < 0.05*.

### Follow-Up and Assessment of Prognosis of All HCC Patients

**Figure 1:**
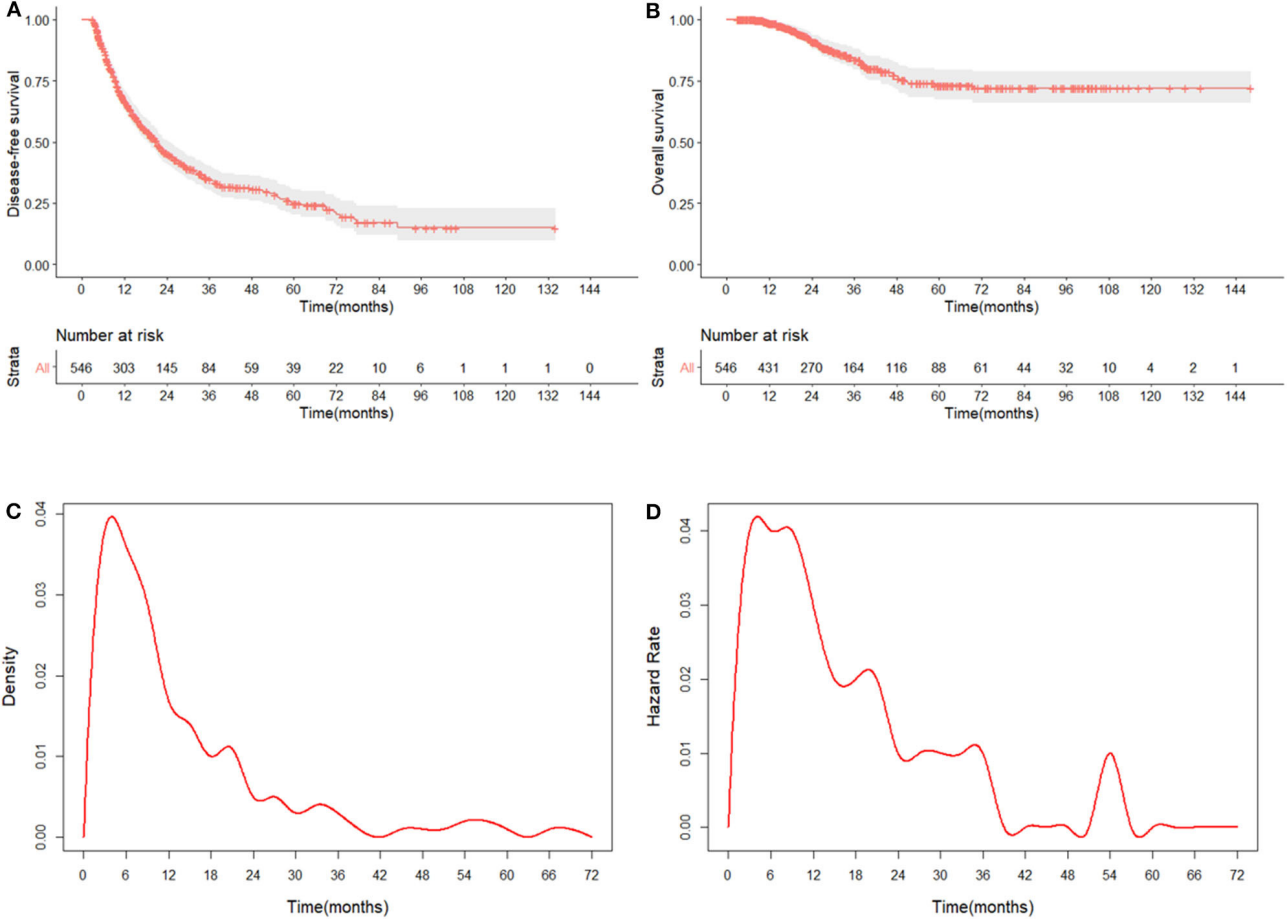
The DFS and OS curves with 95% CIs and risk tables and the recurrence patterns for HCC patients. **(A)** The DFS of 546 patients. The 2- and 5-years DFS rates were 53.0 and 46.0%; **(B)** the OS of 546 patients. The 2- and 5-years OS rates were 88.0 and 87.0%, respectively; **(C)** the probability density plot of recurrence showed that relapse cases centered in the first 2 years after curative treatment; **(D)** the hazard rate of recurrence curve showed that the recurrence hazard peaked during the first 2 years after curative treatment.

Median follow-up time was 23.9 months (range = 3.1–148.3 months), and the median disease-free survival (DFS) and OS were 33.6 and 60.0 months, respectively; 11.9% of patients (65/546) died, with a 2-years OS rate of 88.0%, and the 5-years OS rate was 87.0%; 56.6% of patients (309/546) developed a recurrence, with 2-years DFS rate of 53.0% and 5-years DFS rate of 46.0% (Figures 1A,B). The 1-, 3-, and 5-years survival rates were 99, 97, and 91% in the RS group, and 96, 79, and 72% in the IRS group. Besides, 27.8% of patients (152/546) developed extra-Milan criteria recurrence. In patients with recurrence, the IRS group owned a higher ratio of extra-Milan criteria recurrence than the RS group (*P* = 0.004), 64.6 and 44.7%, respectively. After recurrence, 75% of patients received further treatment, including radical resection (41.5%), local treatment (55.0%), and systemic treatment (3.5%).

From the DFS curve and the probability density plot, we found that 90.0% of patients experienced recurrence within 1 year, and 97.0% of patients experienced recurrence within 2 years (Figures 1A,C). Moreover, the hazard of relapse reached its peak in the first 2 years (Figure 1D). Thus, it makes sense to focus on surveillance during the first 2-years after curative treatments to detect early recurrence at a potentially more treatable stage.

### Univariate and Multivariate Analyses of Prognostic Factors for Recurrence and Survival of All HCC Patients

**Figure 2:**
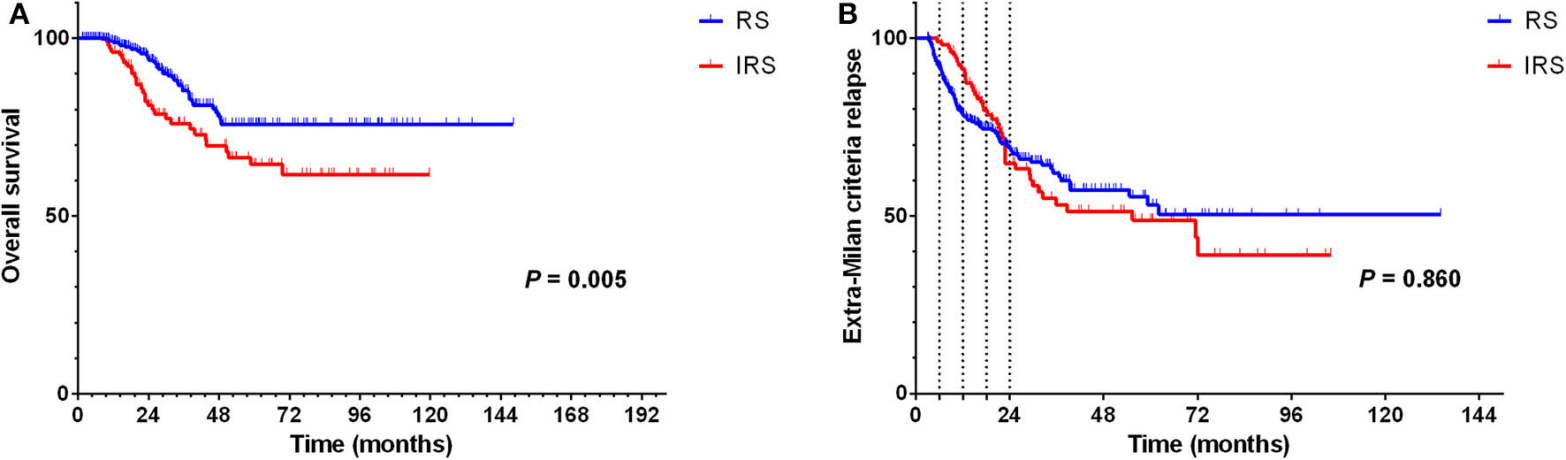
In the RS group, significantly prolonged OS in BCLC stage B HCC patients with CR after radical treatment, but there was no difference between the two groups in terms of the extra-Milan criteria relapse. **(A)** Kaplan–Meier curve for OS of HCC patients stratified by surveillance interval; **(B)** Kaplan–Meier curve for extra-Milan criteria relapse of HCC patients stratified by surveillance interval.

The result of univariate analysis revealed that surveillance interval [*P* = 0.005, HR = 1.981, 95% confidence interval (CI) = 1.227–3.198] (Figure 2A) was prognostic factors for OS, but not for extra-Milan criteria relapse (*P* = 0.860, HR = 0.968, 95% CI = 0.677–1.385) (Figure 2B). Besides, age (*P* = 0.013, HR = 0.498, 95% CI = 0.288–0.863), tumor size (*P* = 0.019, HR = 1.952, 95% CI = 1.116–3.414), and differentiation (*P* = 0.044, HR = 1.552, 95% CI = 1.011–2.381) were prognostic factors for OS (Table 2). In addition, univariate analysis revealed that age (*P* = 0.006, HR = 0.583, 95% CI = 0.398–0.854) was a prognostic factor only for extra-Milan criteria relapse (Table 3).

**Table 2:** Cox proportional hazard regression analysis of patients' overall survival.

**Variable**	**Univariable**		**Multivariable**	
	**HR (95 % CI)**	***P***	**HR (95 % CI)**	***P***
Gender (male/female)	1.239 (0.592–2.589)	0.570		
Age (years) (≤41/>41)	0.498 (0.288–0.863)	**0.013**	0.456 (0.256–0.811)	**0.008**
HBV or HCV (no/yes)	2.150 (0.865–5.5346)	0.099		
Cirrhosis (no/yes)	1.288 (0.759–2.185)	0.348		
AFP (μg/L) (≤400/>400)	1.212 (0.756–1.943)	0.426		
Tumor size (mm) (≤40/>40)	1.952 (1.116–3.414)	**0.019**	2.379 (1.160–4.876)	**0.018**
Multinodular tumor (no/yes)	1.653 (0.965–2.829)	0.067		
One-stage radical treatment (no/yes)	1.381 (0.861–2.215)	0.180		
Therapeutic modalities (surgery/ablation)	0.822 (0.441–1.531)	0.536		
Differentiation (well/moderated/poor)	1.552 (1.011–2.381)	**0.044**	1.509 (0.977–2.331)	0.063
Satellite nodules (no/yes)	1.644 (0.744–3.632)	0.219		
Venous invasion (no/yes)	0.972 (0.530–1.786)	0.928		
Perineural invasion (no/yes)	2.623 (0.363–18.981)	0.339		
Surveillance interval (RS/IRS)	1.981 (1.227–3.198)	**0.005**	1.798 (1.037–3.117)	**0.037**

*The meaning of the bold values provided was p < 0.05*.

**Table 3:** Cox proportional hazard regression analysis of patients' extra-Milan criteria relapse.

**Variable**	**Univariable**		**Multivariable**	
	**HR (95 % CI)**	***P***	**HR (95 % CI)**	***P***
Gender (male/female)	1.248 (0.787–1.978)	0.346		
Age (years) (≤41/>41)	0.583 (0.398–0.854)	**0.006**		
HBV or HCV (no/yes)	0.889 (0.579–1.366)	0.592		
Cirrhosis (no/yes)	1.326 (0.119–1.326)	0.119		
AFP (μg/L) (≤400/>400)	0.767 (0.555–1.061)	0.109		
Tumor size (mm) (≤40/>40)	1.167 (0.839–1.632)	0.359		
Multinodular tumor (no/yes)	1.418 (0.956–2.104)	0.083		
One-stage radical treatment (no/yes)	1.115 (0.809–1.538)	0.506		
Therapeutic modalities (surgery/ablation)	0.764 (0.494–1.181)	0.226		
Differentiation (well/moderated/poor)	0.987 (0.738–1.320)	0.928		
Satellite nodules (no/yes)	1.524 (0.879–2.643)	0.134		
Venous invasion (no/yes)	1.166 (0.812–1.674)	0.406		
Perineural invasion (no/yes)	0.899 (0.126–6.432)	0.916		
Surveillance interval (RS/IRS)	0.968 (0.677–1.385)	0.860		

*The meaning of the bold values provided was p < 0.05*.

Multivariate analysis demonstrated that surveillance interval (*P* = 0.037, HR = 1.798, 95% CI = 1.037–3.117), age (*P* = 0.008, HR = 0.456, 95% CI = 0.256–0.811), and tumor size (*P* = 0.018, HR = 2.379, 95% CI = 1.160–4.876) were independent risk factors for OS (Table 2).

### Univariate and Multivariate Analyses of Prognostic Factors for Survival of HCC Patients With Relapse

**Figure 3:**
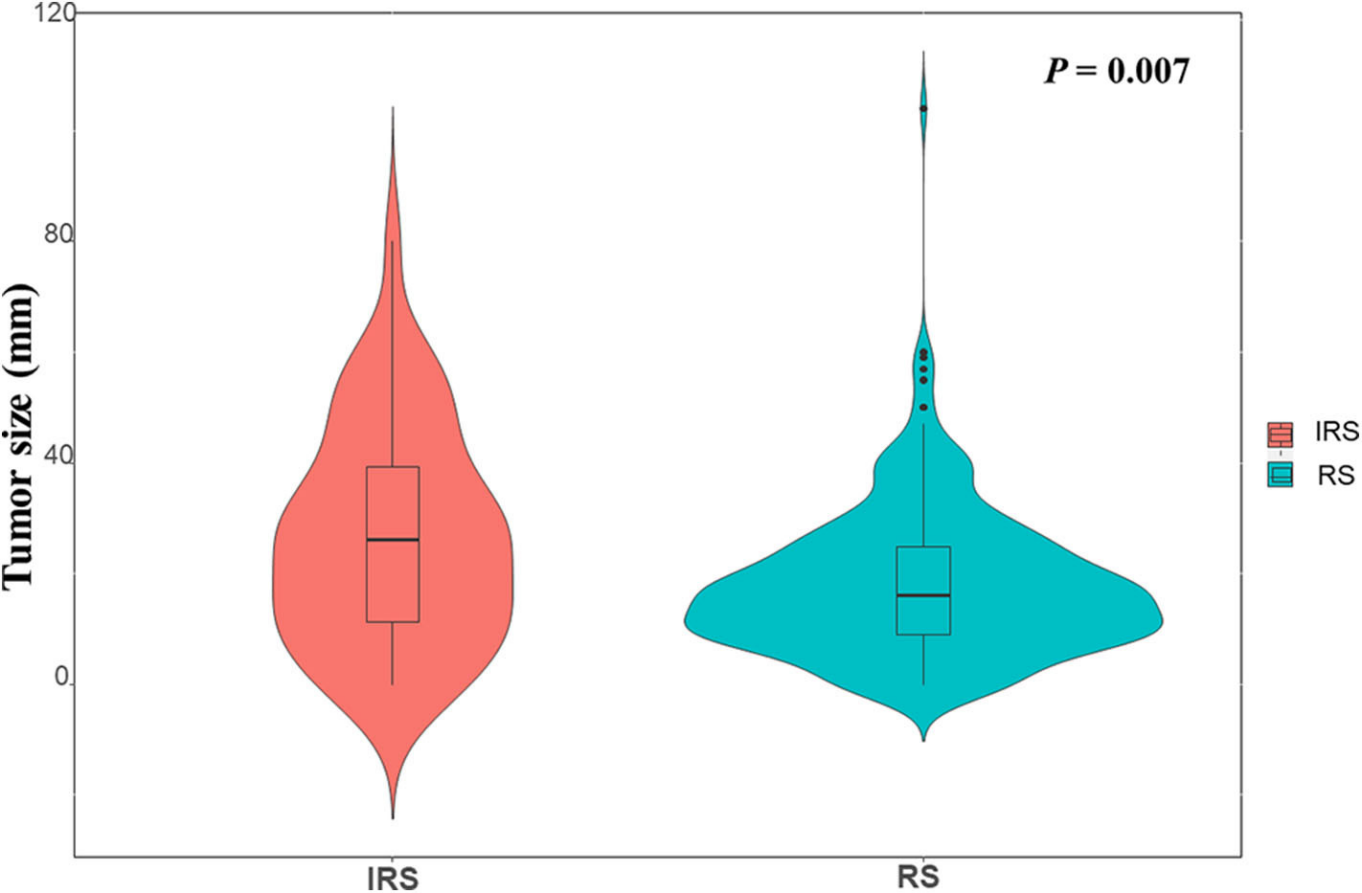
The violin plot indicated that the RS group and the IRS group had significant difference in size of recurrent tumor (*P* = 0.007).

To further assess the association between surveillance interval and survival, further analysis was performed on relapsed patients. The correlation analysis demonstrated that patients in the IRS group owned a higher incidence of extra-Milan criteria recurrence (*P* = 0.004), a larger size of the recurrent tumor (*P* = 0.011), and a higher proportion of multinodular tumors (*P* = 0.003) (Table 4) and less likely to receive secondary treatments after recurrence (*P* = 0.001). Moreover, the violin plot also indicated that the IRS group owned a larger size of the recurrent tumor (Figure 3).

**Table 4:** Correlation between surveillance interval and clinicopathological characteristics with relapsed HCC patients.

**Variable**		**RS (*****n*** **=** **244)**		**IRS (*****n*** **=** **65)**		***P***
		**%**	**No**.	**%**	**No**.	
Extra-Milan criteria relapse	No	55.3	135	35.4	23	**0.004**
	Yes	44.7	109	64.6	42	
AFP (μg/L)	≤400	63.5	155	56.9	37	0.330
	>400	36.5	89	43.1	28	
Relapse location	Local	86.1	210	80.0	52	0.226
	Distant	13.9	34	20.0	13	
Size of recurrent tumor (mm)	≤30	85.7	209	72.3	47	**0.011**
	>30	14.3	35	27.7	18	
Multinodular recurrence	No	79.5	192	61.5	39	**0.003**
	Yes	20.5	55	38.5	26	
Secondary treatment	No	20.6	50	41.5	27	**0.001**
	Yes	79.4	193	58.5	38	

*The meaning of the bold values provided was p < 0.05*.

**Figure 4:**
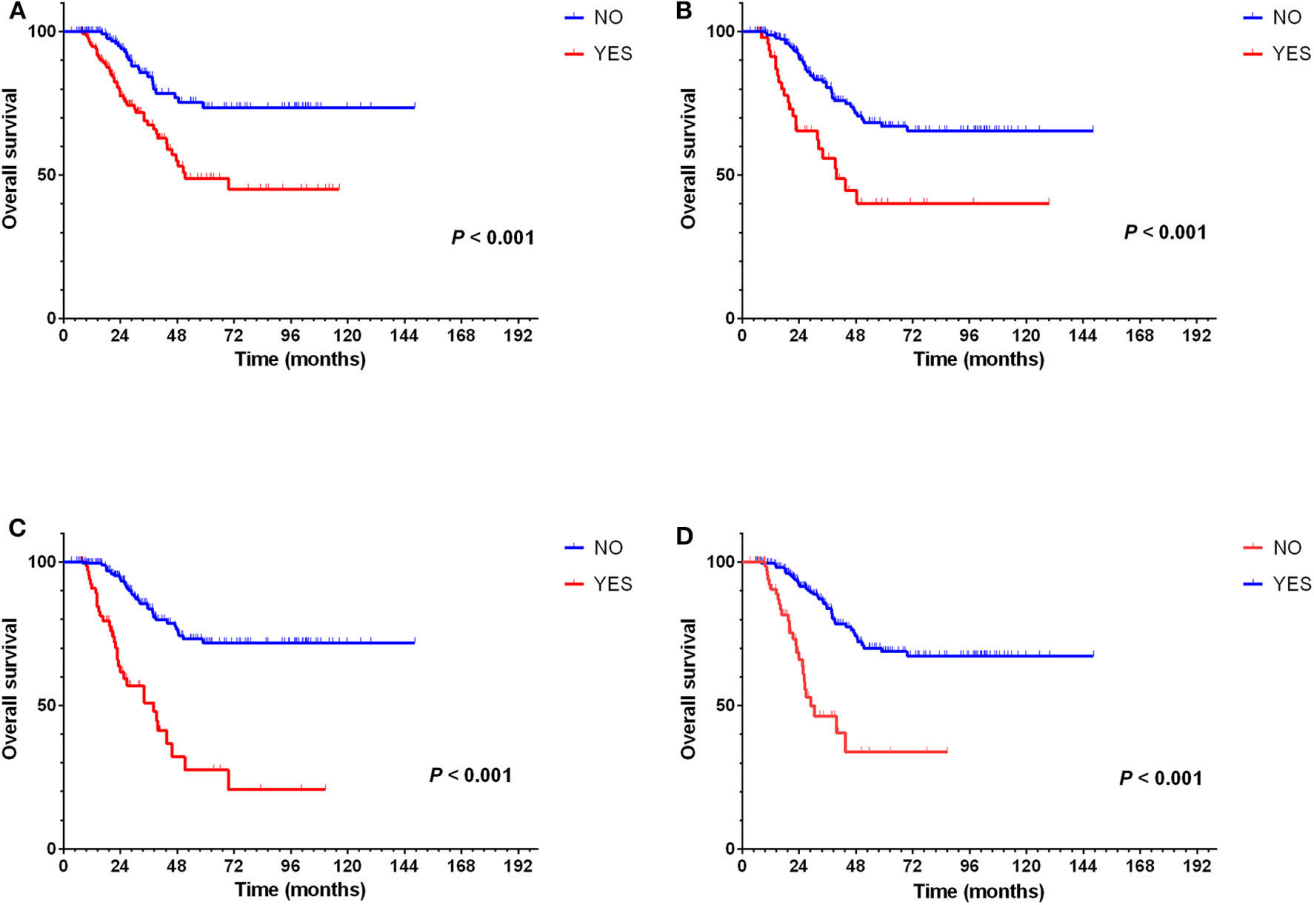
Extra-Milan criteria relapse, size of recurrent tumor, multinodular recurrence, and secondary treatment were prognostic factors for OS but not extra-Milan criteria relapse in relapsed patients. **(A)** Kaplan–Meier curve for OS of relapsed HCC patients stratified by extra-Milan criteria recurrence; **(B)** Kaplan–Meier curve for OS of relapsed HCC patients stratified by size of recurrent tumor; **(C)** Kaplan–Meier curve for OS of relapsed HCC patients stratified by multinodular recurrence; **(D)** Kaplan–Meier curve for OS of relapsed HCC patients stratified by secondary treatment.

**Figure 5:**
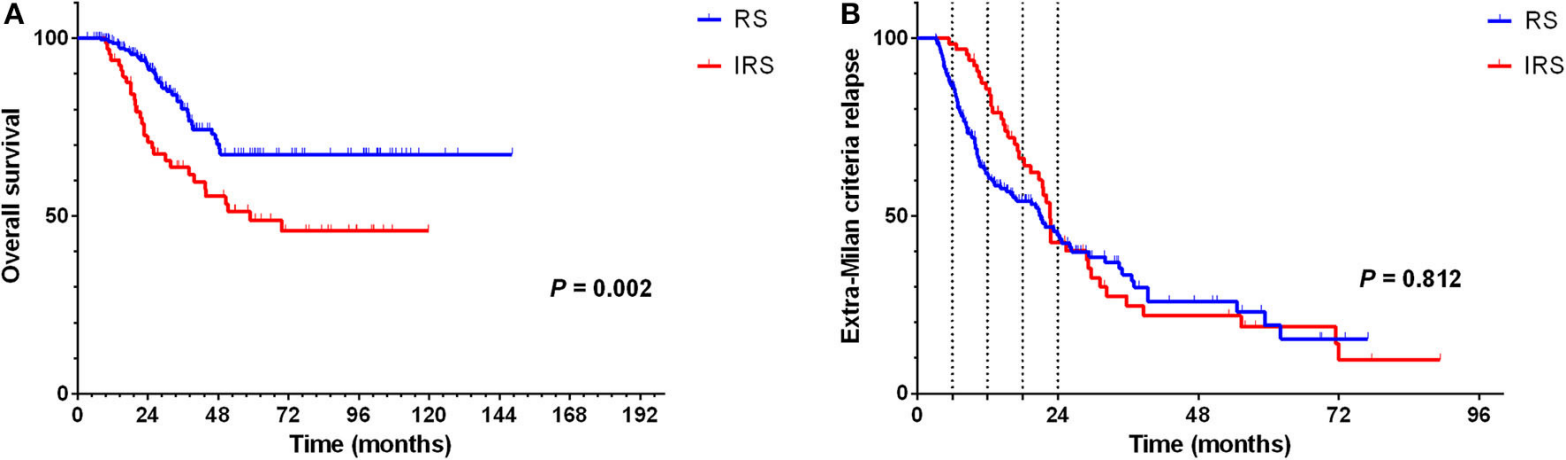
In the RS group, recurrence was significantly prolonged OS in relapsed HCC patients, but there was no difference between the two groups in terms of the extra-Milan criteria relapse. **(A)** Kaplan–Meier curve for OS of relapsed HCC patients stratified by surveillance interval; **(B)** Kaplan–Meier curve for extra-Milan criteria recurrence of relapsed HCC patients stratified by surveillance interval.

Besides, univariate analysis also revealed surveillance interval (*P* = 0.002, HR = 2.160, 95% CI = 1.338–3.488) (Figure 5A), extra-Milan criteria relapse (*P* < 0.001, HR = 2.638, 95% CI = 1.597–4.358) (Figure 4A), size of recurrent tumor (*P* < 0.001, HR = 2.758, 95% CI = 1.661–4.579) (Figure 4B), multinodular recurrence (*P* < 0.001, HR = 4.682, 95% CI = 2.903–7.552) (Figure 4C), and secondary treatment (*P* < 0.001, HR = 0.261, 95% CI = 0.155–0.439) (Figure 4D) were prognostic factors for OS in relapsed patients (Table 5). Multivariate analysis demonstrated that extra-Milan criteria relapse (*P* = 0.038, HR = 1.782, 95% CI = 1.032–3.077) and secondary treatment (*P* < 0.001, HR = 0.335, 95% CI = 0.193–0.581) were independent risk factors for OS (Table 5).

**Table 5:** Cox proportional hazard regression analysis of relapsed patients' overall survival.

**Variable**	**Univariable**		**Multivariable**	
	**HR (95 % CI)**	***P***	**HR (95 % CI)**	***P***
Extra-Milan criteria relapse (no/yes)	2.638 (1.597–4.358)	** <0.001**	1.782 (1.032–3.077)	**0.038**
AFP (μg/L) (≤400/>400)	0.912 (0.562–1.480)	0.710		
Relapse location (local/distant)	1.055 (0.688–1.618)	0.805		
Size of recurrent tumor (mm) (≤30/>30)	2.758 (1.661–4.579)	** <0.001**		
Multinodular recurrence (no/yes)	4.682 (2.903–7.552)	** <0.001**		
Secondary treatment (no/yes)	0.261 (0.155–0.439)	** <0.001**	0.335 (0.193–0.581)	** <0.001**
Surveillance interval (RS/IRS)	2.160 (1.338–3.488)	**0.002**	1.309 (0.777–2.207)	0.312

*The meaning of the bold values provided was p < 0.05*.

### Comparison of Surveillance Interval and 0–18 Months Extra-Milan Criteria Relapse of All HCC Patients

**Figure 6:**
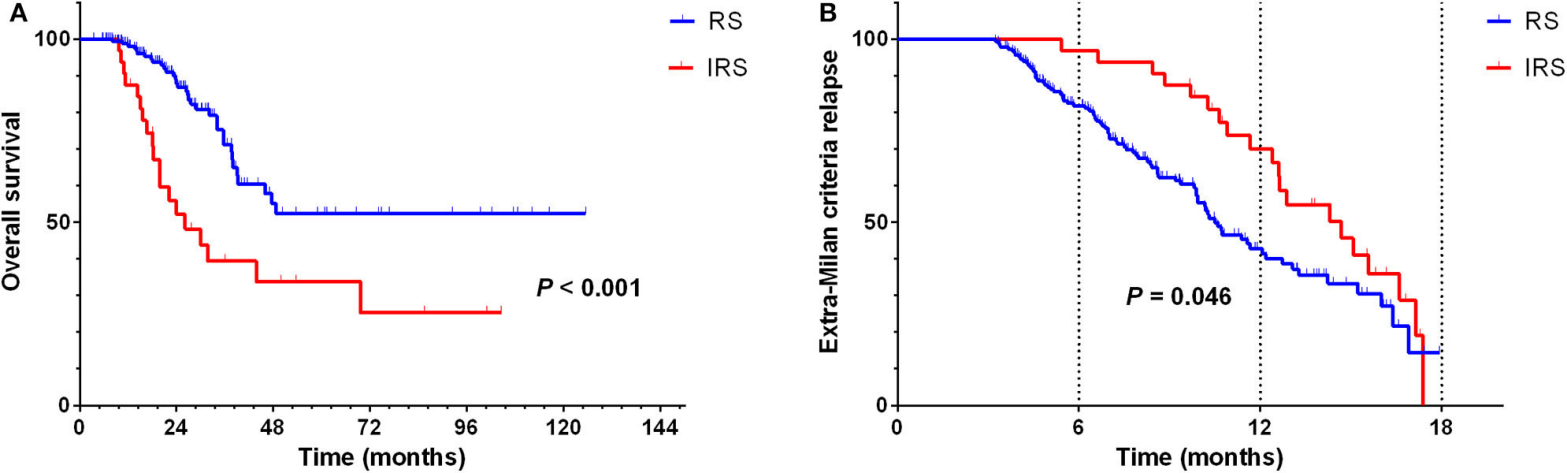
In the RS group, extra-Milan criteria recurrence was detected earlier and significantly prolonged OS in 0–18 months relapsed HCC patients. **(A)** Kaplan–Meier curve for OS of 0–18 months relapsed HCC patients stratified by surveillance interval; **(B)** Kaplan–Meier curve for extra-Milan criteria recurrence of 0–18 months relapsed HCC patients stratified by surveillance interval.

According to the hazard rate curve that the recurrence risk of BCLC stage B patients with CR was still high at 0–24 months (Figure 1D). Moreover, although the K-M curve showed no difference between the RS and IRS groups for extra-Milan criteria relapse in HCC patients (Figure 2B) and HCC patients relapse (Figure 5B), the interval between 0 and 18 months of surveillance also appeared to be associated with extra-Milan criteria relapse (Figure 5B). In the further analysis of patients with extra-Milan criteria relapse in 0–18 months, we found that the RS group could earlier detect extra-Milan criteria relapse (*P* = 0.046, HR = 0.602, 95% CI = 0.366–0.991) (Figure 6B), which significantly prolonged OS (*P* < 0.001, HR = 2.893, 95% CI = 1.647–5.082) (Figure 6A). Based on this, we further analyzed the surveillance interval of 0–6, 6–12, 12–18, and 18–24 months.

**Figure 7:**
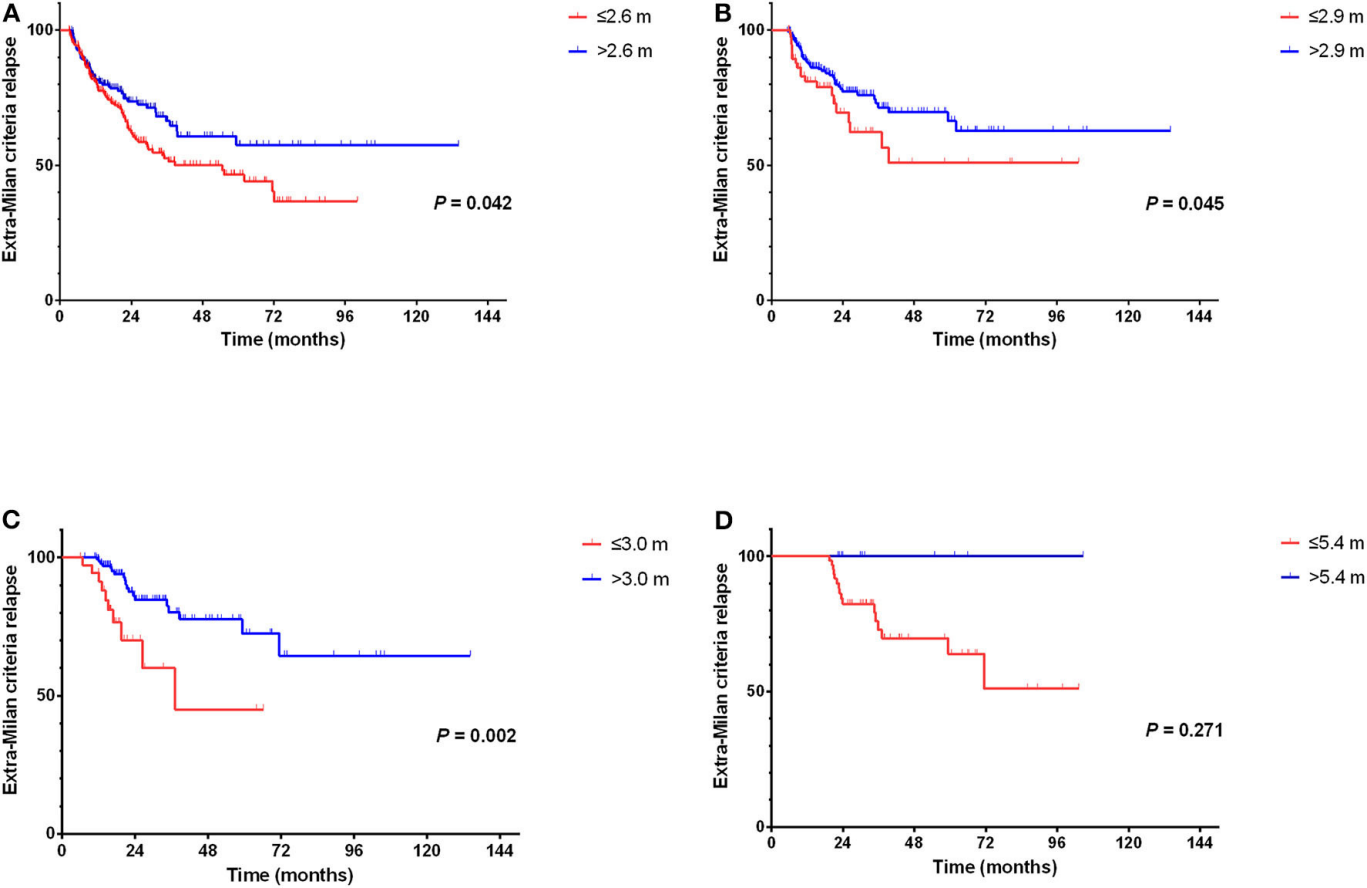
Comparison of surveillance interval and extra-Milan criteria relapse. **(A)** Patients with an average surveillance interval ≤2.6 months within 0–6 months could earlier detect extra-Milan criteria relapse; **(B)** patients with an average surveillance interval ≤2.9 months within 6–12 months could earlier detect extra-Milan criteria relapse; **(C)** patients with an average surveillance interval ≤3.0 months within 6–12 months could earlier detect extra-Milan criteria relapse; **(D)** there was no significant difference between the average surveillance interval within 18–24 months.

We found that patients with an average surveillance interval ≤2.6 months within 0–6 months could earlier detect extra-Milan criteria relapse (*P* = 0.042, HR = 0.713, 95% CI = 0.515–0.988) (Figure 7A). In addition, patients with an average surveillance interval ≤2.9 months within 6–12 months (*P* = 0.045, HR = 0.593, 95% CI = 0.356–0.989) and an average surveillance interval ≤3 months within 12–18 months (*P* = 0.002, HR = 0.299, 95% CI = 0.137–0.654) could earlier detect extra-Milan criteria relapse (Figures 7B,C). However, there was no significant difference between the average surveillance interval within 18–24 months (*P* = 0.271, HR = 0.038, 95% CI = 0.000–12.896) (Figure 7D).

## Discussion

Currently, the European Society for Medical Oncology proposes that follow-up of patients who underwent radical treatments (resection or ablation) should consist of the clinical evaluation of liver decompensation and the early detection of recurrence by dynamic CT or MRI studies every 3 months during the 1st year and surveillance every 6 months thereafter (10). But the National Comprehensive Cancer Network offers a different view, recommending continuous surveillance every 3–6 months, for 2 years, and then every 6–12 months (19). However, these two guidelines are not sufficient to guide clinical practice, in which the follow-up strategies of the two clinical guidelines are quite different in terms of the surveillance interval, not for specific patients. Also, the guidelines do not specifically recommend surveillance intervals for BCLC stage B HCC patients with CR, which were more complicated and had relapses earlier than did those of BCLC stage A. Although patients are recommended for surveillance according to clinical guidelines, in the real world, for a variety of reasons, patients cannot fully follow the guidelines for surveillance strategies. Therefore, the impact of IRS in the real world on patient survival is unclear.

Previous studies have indicated that earlier identification of disease may facilitate patient eligibility for investigational studies or other forms of treatment (19, 20). HY K et al. demonstrated that the detection of small HCC eligible for curative treatment is increased by frequent surveillance (16, 18). Besides, patients in the RS group were diagnosed at earlier stages than the IRS or non-surveillance groups, which had more chance for curative treatments (18). Moreover, AA M et al. also reported that a long surveillance interval compromises OS in high-risk patients who underwent curative thermal ablation for HCC within the Milan criteria (21). Besides, other tumors have the same results for which more intensive surveillance after surgery for esophagogastric adenocarcinoma, colorectal cancer, and non–small cell lung cancer translates into improved survival (22–25). Although there is no high-level evidence, the cutoff of 2 years has been adopted to grossly classify early and late recurrences (14, 26). In our result, we also found that the vast majority of patients experienced recurrence within 2 years. Other than that, we also proved that RS owned a lower incidence of extra-Milan criteria relapse and smaller and fewer tumors at recurrence than those of IRS group, which contributed to the prolonged OS. Thereby, the average surveillance interval for patients with BCLC stage B HCC who achieved CR should not exceed 4.3 months during the first 2 years' follow-up.

Over the past 20 years, the Milan criteria have been highly successful in selecting patients for good long-term survival and remain the criteria for potential transplant candidates for HCC (27). It is important to identify the possible predictive factors of within and extra-Milan criteria recurrences after radical treatments (28). Early diagnosis of extra-Milan criteria recurrence can enable patients to receive a more timely intervention after recurrence and control the development of tumors. In our study, we also found that the RS group could earlier detect extra-Milan criteria relapse and significantly prolonged OS in 0–18 months relapsed patients. Moreover, during 0–6, 6–12, and 12–18 months of the initial 18 months after CR, individualized surveillance intervals that no more than 3 months were required to reduce the incidence of extra-Milan criteria relapse. The interval of surveillance according to current guidelines is therefore insufficient, especially 12–18 months after CR.

As mentioned above, despite this study having many clinical implications, we should be clear that it is a retrospective study with its limitations. First, our study was conducted in a single center. The collection of multicenter data to expand the sample size is the next step that needs to be done. Moreover, the follow-up strategy of patients in different stages after radical operation needs to be further explored. Finally, RS could detect tumor recurrence at an early stage and prolong the survival of patients, which requires further clinical trials to verify it.

In conclusion, our results demonstrated that the surveillance interval for BCLC stage B HCC patients with CR after curative treatment should not exceed 4.3 months during the first 2 years' follow-up. Besides, during 0–6, 6–12, and 12–18 months of the initial 18 months after CR, individualized surveillance intervals of no more than 3 months were required to reduce the incidence of extra-Milan criteria relapse.

## Data Availability Statement

The raw data supporting the conclusions of this article will be made available by the authors, without undue reservation.

## Ethics Statement

The studies involving human participants were reviewed and approved by the Institutional Review Board of Sun Yat-sen University Cancer Center. Written informed consent to participate in this study was provided by the participants or their legal guardian/next of kin.

## Author Contributions

WF: study conception. YW, LS, and HQ: analysis and interpretation of data. All authors: acquisition of data, drafting of manuscript, and final approval.

## Conflict of Interest

The authors declare that the research was conducted in the absence of any commercial or financial relationships that could be construed as a potential conflict of interest.

## Abbreviations

HCC: hepatocellular carcinoma
CR: complete remission
BCLC: Barcelona Clinic Liver Cancer
RS: regular surveillance
IRS: irregular surveillance
HBV: hepatitis B virus
HCV: hepatitis C virus
DFS: disease-free survival
OS: overall survival
TACE: transarterial chemoembolization
CT: multidetector computed tomography
MRI: magnetic resonance imaging
AFP: α-fetoprotein
HR: hazard ratio
CI: confidence interval.
